# Sequenced BAC anchored reference genetic map that reconciles the ten individual chromosomes of *Brassica rapa*

**DOI:** 10.1186/1471-2164-10-432

**Published:** 2009-09-15

**Authors:** HyeRan Kim, Su Ryun Choi, Jina Bae, Chang Pyo Hong, Seo Yeon Lee, Md Jamil Hossain, Dan Van Nguyen, Mina Jin, Beom-Seok Park, Jea-Wook Bang, Ian Bancroft, Yong Pyo Lim

**Affiliations:** 1 Plant Genomics Institute, Chungnam National University, Daejeon, 305-764 Korea; 2 Department of Horticulture, Chungnam National University, Daejeon, 305-764 Korea; 3 National Institute of Agricultural Biotechnology, Rural Development Administration, Suwon, 441-707 Korea; 4 Department of Biology, Chungnam National University, Daejeon, 305-764 Korea; 5 Department of Crop Genetics, John Innes Centre, Norwich Research Park, Colney, Norwich NR4 7UH, UK; 6 Macrogen Inc., Seoul 153-023, Korea

## Abstract

**Background:**

In view of the immense value of *Brassica rapa *in the fields of agriculture and molecular biology, the multinational *Brassica rapa *Genome Sequencing Project (BrGSP) was launched in 2003 by five countries. The developing BrGSP has valuable resources for the community, including a reference genetic map and seed BAC sequences. Although the initial *B. rapa *linkage map served as a reference for the BrGSP, there was ambiguity in reconciling the linkage groups with the ten chromosomes of *B. rapa*. Consequently, the BrGSP assigned each of the linkage groups to the project members as chromosome substitutes for sequencing.

**Results:**

We identified simple sequence repeat (SSR) motifs in the *B. rapa *genome with the sequences of seed BACs used for the BrGSP. By testing 749 amplicons containing SSR motifs, we identified polymorphisms that enabled the anchoring of 188 BACs onto the *B. rapa *reference linkage map consisting of 719 loci in the 10 linkage groups with an average distance of 1.6 cM between adjacent loci. The anchored BAC sequences enabled the identification of 30 blocks of conserved synteny, totaling 534.9 cM in length, between the genomes of *B. rapa *and *Arabidopsis thaliana*. Most of these were consistent with previously reported duplication and rearrangement events that differentiate these genomes. However, we were able to identify the collinear regions for seven additional previously uncharacterized sections of the A genome. Integration of the linkage map with the *B. rapa *cytogenetic map was accomplished by FISH with probes representing 20 BAC clones, along with probes for rDNA and centromeric repeat sequences. This integration enabled unambiguous alignment and orientation of the maps representing the 10 *B. rapa *chromosomes.

**Conclusion:**

We developed a second generation reference linkage map for *B. rapa*, which was aligned unambiguously to the *B. rapa *cytogenetic map. Furthermore, using our data, we confirmed and extended the comparative genome analysis between *B. rapa *and *A. thaliana*. This work will serve as a basis for integrating the genetic, physical, and chromosome maps of the BrGSP, as well as for studies on polyploidization, speciation, and genome duplication in the genus *Brassica*.

## Background

*Brassica *is a model system for studying polyploidization and speciation since all the species in this genus have descended from a common hexaploid ancestor [1-6]. In addition, *Brassica *species share an ancestor with *Arabidopsis*, implying a similar basic genome, and thereby providing sequence-level colinearity between the two genera, particularly in euchromatic regions [7-9]. This relationship highlights the feasibility of utilizing the accumulated *Arabidopsis *information for the study of *Brassica *species. The genus *Brassica *includes economically important crop taxa with a wide range of morphologies, such as Chinese cabbage, mustard, cabbage, broccoli, oilseed rape, and other leafy vegetables. These taxa are classified into six genome types (AA, n = 10; BB, n = 8; CC, n = 9; AABB, n = 18; AACC, n = 19; BBCC, n = 17) according to the six representative species (AA, *B. rapa*; BB, *B. nigra*; CC, *B. oleracea*; AABB, *B. juncea*; AACC, *B. napus*; BBCC, *B. carinata*), and the genomic relationships of these taxa are well defined in U's triangle [10].

In view of the enomous value of *Brassica *in the fields of agriculture and molecular biology, genome sequencing projects have been proposed for each of the three diploid genomes [11]. In order to better understand the A genome of *Brassica *and to take advantage of the colinearity between this genome and the *Arabidopsis *genome sequence, the multinational *Brassica rapa *Genome Sequencing Project (BrGSP) was launched in 2003 by scientists from five countries (Korea, Canada, the United Kingdom, China, and Australia) for sequencing *B. rapa *ssp. *pekinensis *cv. Chiifu-401-42 using a BAC-by-BAC approach [11]. The initial objective of the BrGSP was to sequence the gene space of *B. rapa*, which represents approximately 330 Mb of its genome [12], at a Phase II quality level, whereby BACs would be sequenced to a single ordered and oriented contig, but with allowance for some gaps and ambiguous bases [13]. The Korean group of the BrGSP [Korean *Brassica *Genome Project (KBGP)] sequenced 521 *B. rapa *BACs selected to represent genomic regions collinear with the majority of the euchromatic regions of the *A. thaliana *genome [12]. These clones serve as "seed" BACs for the BrGSP, from which chromosome-scale sequencing is being initiated. The developing BrGSP has valuable resources for the community, including three BAC libraries [2,14], 200,017 BAC end sequences [15], 129,928 EST sequences (by April 2008), and an initial-version reference genetic map [16]. To date, 631 BAC sequences, representing approximately 75.3 Mb, have been made public [13].

The initial reference linkage map of *B. rapa *was constructed with 556 markers (278 AFLPs; 235 SSRs; 25 RAPDs; and a total of 18 ESTPs, STSs, and CAPSs) based on 78 doubled haploid lines (CKDH line) derived from an anther culture of the F_1 _of a cross between diverse Chinese cabbage (*B. rapa *ssp. *pekinensis*) inbred lines; "Chiifu-401-42" (C) and "Kenshin-402-43" (K) [16]. Ten linkage groups, designated as A1-A10 according to the common nomenclature of the *B. napus *reference linkage maps [17], served as a reference for the BrGSP. However, there remained ambiguity in reconciling the linkage groups with the 10 *B. rapa *chromosomes characterized using cytogenetic approaches.

The *B. rapa *chromosomes have been extensively studied by karyotyping based on morphometric measurements of mitotic metaphase chromosomes [18-21]. The definitive identification of each of the individual chromosomes has been problematic because some are small-sized or similar. Recently, using fluorescence *in situ *hybridization (FISH), six of the 10 chromosomes were distinguished unambiguously based on the chromosomal position of repetitive sequences, such as 45S rDNA, 25S rDNA, 5S rDNA, and centromeric repeats. However, this technique can be impractical in that multiple FISHs are required to distinguish these six chromosomes, and it is unable to distinguish the remaining four chromosomes [14,22-25]. Consequently, the BrGSP assigned each of the linkage groups (A1-A10) to the project members as chromosomal substitutes for sequencing [11].

In this study, we developed a second generation *B. rapa *reference linkage map, aligned unambiguously with the cytogenetic map of *B. rapa*. We also used our data to confirm and extend the comparative genome analysis of *B. rapa *and *A. thaliana*.

## Results

### Construction of the second generation reference linkage genetic map

**Figure 1:**
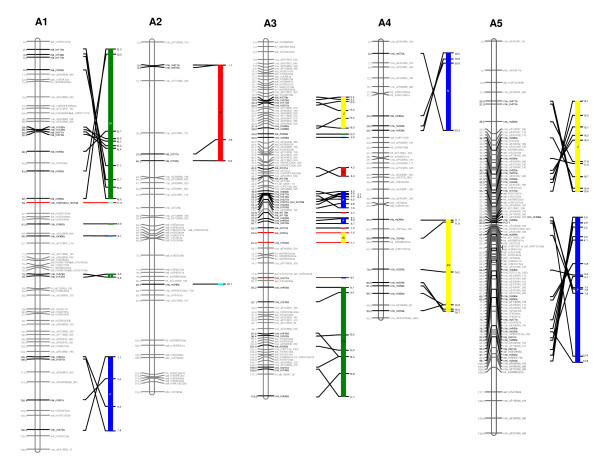
**The second generation reference genetic map of *B. rapa *and its alignment onto the *Arabidopsis *genome sequence (linkage groups A1, A2, A3, A4 and A5)**. Cumulative recombination distances are shown to the left and marker loci to the right of the linkage groups. SSR markers developed from the BAC sequences are designated in bold strokes. The correspondence between the SSR markers and the BAC clones is given in Additional file 2. The colored bars to the right of the linkage groups indicate *Arabidopsis *chromosomes (chromosome 1: light blue; chromosome 2: yellow; chromosome 3: dark blue; chromosome 4: green; chromosome 5: red), representing the synteny blocks between the two genomes. The synteny blocks identified by Schranz et al. [28] are embedded in the colored bars. New blocks proposed in this study, not identified by Schranz et al. [28], are marked with red lines. The numbers to the right of the colored bars indicate aligned positions on *Arabidopsis *chromosomes in megabase pairs (Mb). *Arabidopsis *chromosomes are not to scale.

**Figure 2:**
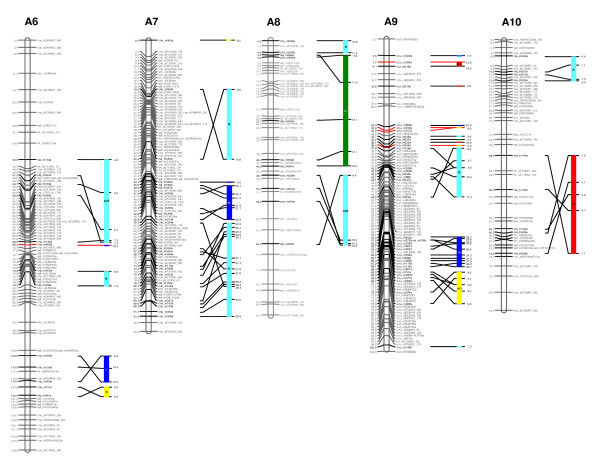
**The second generation reference genetic map of *B. rapa *and its alignment onto the *Arabidopsis *genome sequence (linkage groups A6, A7, A8, A9 and A10)**. Cumulative recombination distances are shown to the left and marker loci to the right of the linkage groups. SSR markers developed from the BAC sequences are designated in bold strokes. The correspondence between the SSR markers and the BAC clones is given in Additional file 2. The colored bars to the right of the linkage groups indicate *Arabidopsis *chromosomes (chromosome 1: light blue; chromosome 2: yellow; chromosome 3: dark blue; chromosome 4: green; chromosome 5: red), representing the synteny blocks between the two genomes. The synteny blocks identified by Schranz et al. [28] are embedded in the colored bars. New blocks proposed in this study, not identified by Schranz et al. [28], are marked with red lines. The numbers to the right of the colored bars indicate aligned positions on *Arabidopsis *chromosomes in megabase pairs (Mb). *Arabidopsis *chromosomes are not to scale.

Of the 749 SSR motif-containing amplicons designed from 367 sequenced BACs, 311 (41.5%) showed polymorphism between Chiifu and Kenshin (Additional file 1). Many amplicons designed from *Brassica *sequences produce multiple bands as a consequence of the extensive genome duplication observed in these species. Anchorage of sequenced BAC clones to linkage maps therefore requires matching of the size of the PCR product from the BAC (derived from Chiifu genomic DNA) with the size of the polymorphic band amplified from genomic DNA (the Chiifu allele). This validation was conducted for all polymorphic markers. The polymorphic PCR product from the genomic DNA was found to match the BAC-derived product for 191 markers (61.4%), all of which were designed from 188 different BAC clones. There were several instances of the experimentally determined band being greater in size than was expected from the BAC sequences. We attribute these differences to sequencing errors. The 191 BAC-anchoring markers were added to the initial version of the *B. rapa *linkage map to produce the second generation linkage map (Fig. 1, Fig. 2). This map now anchors 188 sequenced BAC clones to the *B. rapa *linkage map.

The linkage map contained 719 loci, comprising 267 AFLP, 411 SSR, 24 RAPD, 8 STS, 7 ESTP, and 2 CAPS markers, assigned to the 10 A genome linkage groups (A1-A10) of *Brassica *species following established nomenclature [26]. The total length of the map was 1,123.3 cM with an average distance of 1.6 cM between adjacent loci (Table 1). The length of the linkage groups ranged from 91.3 cM (A10) to 138.5 cM (A06), and the number of markers in the 10 linkage groups ranged from 38 (A4) to 104 (A3). In total, 24.3% of the markers were mapped at the same loci or at less than 1-cM intervals. The average distance between adjacent loci was greatest for A2 (2.8 cM) and least for A7 (1.0 cM). Two gaps of greater than 10 cM were present (A2), measuring 13.5 cM and 17.7 cM, respectively.

**Table 1:** Salient features of the sequenced BAC anchored second generation reference genetic map of *B. rapa*


**Linkage group**	**No. of total markers**	**No. of SSR markers (%)**	**Average distance between two loci (cM)**	**No. of anchored BACs**	**No. of markers with 1 cM intervals**^a^	**No. of gaps**^b^	**Length (cM)**

A1	67	46 (69%)	2.0	20	56	0	136.9
A2	42	27 (64%)	2.8	4	35	2	118.5
A3	104	64 (62%)	1.1	35	80	0	118.6
A4	38	21 (55%)	2.4	11	36	0	92.3
A5	97	50 (52%)	1.3	25	64	0	130.8
A6	86	40 (47%)	1.6	13	68	0	138.5
A7	98	49 (50%)	1.0	24	64	0	98.4
A8	47	24 (51%)	2.0	11	38	0	93.2
A9	91	65 (71%)	1.2	36	60	0	104.8
A10	49	25 (51%)	1.9	9	43	0	91.3

Total/avg	719	411 (57%)	1.6	188	544 (76%)	2	1,123.3

^a^Adjacent markers > 1 cM.

^b^Distance between adjacent markers ≥ 10 cM.

### Aligning the linkage map with the *Arabidopsis *genome sequence

In order to estimate the coverage of the linkage map with respect to the *Arabidopsis *genome, we aligned the sequences of the anchored BACs to the *Arabidopsis *genome sequence (Fig. 1, Fig. 2). Of the 188 anchored BAC clones, 184 (representing 187 loci) could be aligned. The aligned regions represent 534.9 cM of the *B. rapa *linkage map and 60.5 Mb of the *Arabidopsis *genome sequence (Fig. 1, Fig. 2, Table 2, Additional file 2).

**Table 2:** Summary of the synteny blocks between the *B. rapa *genetic map and the *Arabidopsis *genome sequence deduced from BAC alignment


***Arabidopsis *chromosome**	**No. of *B. rapa *BACs aligned**	***B. rapa *linkage groups aligned (cM)**	**Total length of *B. rapa *linkage groups aligned**	**Total length of *Arabidopsis *genome aligned**^a^	**Length of *Arabidopsis *genome covered by the alignment ** **(% coverage)**	**Total length of *Arabidopsis *chromosome**^b^

1	43	A6 (32.0), A7 (49.1), A8 (27.3), A9 (12.1), A10 (8.5)	129.0 cM	21.6 Mb	13.7 Mb (45%)	30.4 Mb
2	36	A3 (12.5), A4 (30.5), A5 (35.1), A6 (2.8), A9 (7.9)	88.7 cM	17.9 Mb	10.0 Mb (51%)	19.7 Mb
3	59	A1 (24.4), A3 (5.7), A4 (25.7), A5 (38.8), A6 (8.5), A7 (9.6), A9 (10.9)	123.6 cM	33.4 Mb	16.9 Mb (72%)	23.5 Mb
4	30	A1 (50.4), A3 (36.5), A8 (37.7)	124.6 cM	24.2 Mb	10.1 Mb (54%)	18.6 Mb
5	16	A2 (32.2), A3 (2.4), A9 (1.4), A10 (33.1)	69.1 cM	14.6 Mb	9.9 Mb (37%)	27.0 Mb

Total	184		534.9 cM (48%)	111.7 Mb	60.5 Mb (51%)	119.2 Mb

^a^Details of redundantly represented regions in the *B. rapa *genome are in Table 5.

^b^[51]

We detected 30 blocks of conserved synteny, as defined by two or more adjacent anchored *B. rapa *BACs aligned to the corresponding region of the *Arabidopsis *genome identified in the earlier maps for A genomes [27,28] (Fig. 1, Fig. 2, Table 3). If the extended positions were detected within our defined blocks of the *B. rapa *map (Table 3), they were considered extensions of the previously reported blocks (A-X). Blocks G, S, and H (in blocks 9, 25, and 28) were newly identified in the *B. rapa *linkage groups A3, A9, and A9, respectively, compared with the A genome map of *B. napus *[27,28]. Blocks G and H in the linkage groups of A3 and A9 were recognized in the A genome of *B. juncea *[29], whereas block S in A9 (in block 25) appeared to be unique to the *B. rapa *genome. The longest blocks were 59.9-98.7 cM on A5, aligned to 10.8 Mb of *Arabidopsis *chromosome 3, and 2.7-52.7 cM on A1, aligned to 6.2 Mb of *Arabidopsis *chromosome 4. On average (based on each of the 30 blocks of conserved synteny), we estimate that 1 cM of the *B. rapa *reference genetic map aligns to 341 kb of the *Arabidopsis *genome sequence. However, the large standard deviation (513 kb) indicates that this relationship varies greatly across the aligned genome segments. This alignment updated the initial version of the *B. rapa *linkage map [16].

**Table 3:** Summary of the synteny blocks between the *B. rapa *second generation reference genetic map and the *Arabidopsis *genome sequence


**Synteny block**	***B. rapa***				***Arabidopsis***				**Aligned *Arabidopsis***	**Corresponding genome block identified by**	
									**chromosome **		
	**Aligned position**			**Aligned **	**Aligned position**			**Aligned **	****length(Kb)****		
				**length (cM)**				**length (cM)**	**per 1 cM *of B. rapa***		
	**Linkage group**	**From (cM)**	**To (cM)**		**Chromosome**	**From (Mb)**	**To (Mb)**			**Schranz et al. 2006 **[29]	**Parkin et al. 2005 **[28]

Block01^a^	A01	2.7	52.7	50.0	4	12.3	18.6	6.2	125	U	C4B
Block02		78.2	78.7	0.4	4	8.5	8.8	0.3	683	T	C4B'
Block03		105.9	130.3	24.4	3	1.1	7.5	6.4	260	F	C3A

Block04	A02	8.0	40.2	32.2	5	1.1	5.9	4.8	150	R	C5A

Block05^a^	A03	19.6	28.8	9.2	2	12.9	18.6	5.7	618	J	C2C
Block06		42.6	45.0	2.4	5	4.2	4.5	0.3	142	R	C5A
Block07		49.9	52.8	2.9	3	4.5	7.8	3.3	1,168	F	C3A
Block08		57.2	60.1	2.9	3	8.1	8.9	0.8	268	F	C3A
Block09^a^		63.9	67.2	3.2	2	1.6	2.2	0.6	183	G	C2A
Block10^a^		82.2	118.6	36.5	4	8.7	17.8	9.1	249	T, U	C4B', C4B

Block11^a^	A04	4.0	29.7	25.7	3	19.5	23.4	3.9	152	N	C3D
Block12		59.4	89.9	30.5	2	11.7	16.3	4.5	148	I, J	C2B, C2C

Block13^a^	A05	20.0	55.1	35.1	2	14.1	19.7	5.6	160	J	C2C
Block14^a^		59.9	98.7	38.8	3	2.5	13.3	10.8	279	F	C3A

Block15^a^	A06	40.3	68.2	27.9	1	3.5	7.4	3.9	140	A, B	C1A, C1B
Block16^a^		74.2	78.3	4.1	1	6.8	7.3	0.5	118	B	C1B
Block17		106.6	115.0	8.5	3	9.8	11.1	1.4	163	L	C3B
Block18		117.2	120.0	2.8	2	0.2	1.0	0.9	308	K	C2A

Block19^a^	A07	19.0	34.3	15.3	1	8.8	10.9	2.1	135	B	C1B
Block20^a^		45.3	54.8	9.6	3	20.1	22.8	2.6	276	N	C3D
Block21		59.5	93.3	33.8	1	25.5	30.0	4.5	133	E	C1E

Block22	A08	0.0	4.0	4.0	1	10.8	11.1	0.3	63	B	C1B
Block23^a^		4.9	42.6	37.7	4	9.9	18.4	8.5	225	U	C4B
Block24^a^		45.7	69.0	23.2	1	4.4	10.6	6.1	264	A, B	C1A, C1B

Block25^a^	A09	7.7	9.1	1.4	5	15.9	19.8	3.8	2,796	S, V	C5C, C5D
Block26		37.5	49.6	12.1	1	8.4	11.2	2.8	230	B	C1B
Block27		57.3	68.1	10.9	3	18.7	22.8	4.2	383	N	C3D
Block28		69.2	77.0	7.9	2	9.0	9.6	0.6	77	H, I	C2A, C2B

Block29	A10	5.3	13.8	8.5	1	0.6	2.1	1.5	173	A	C1A
Block30		38.9	72.0	33.1	5	1.6	7.2	5.6	169	R	C5A

^a^Blocks expanded compared to the previous reports [27,28]

Most of the alignments between the linkage map and the *Arabidopsis *genome sequence were consistent with collinearity blocks previously inferred using sequenced markers with homology to single *Arabidopsis *genes [27,28]. However, we identified the collinear regions for seven additional previously uncharacterized sections of the A genome by a single aligned BAC, and confirmed three of these by mapping a second marker. The number and ranges of the *Arabidopsis *gene models for which collinear homologous sequences could be identified were determined by using BAC annotations of the BrGSP [30], and these are shown in Table 4.

**Table 4:** Further enrichment of previously defined collinearity between the *Brassica *A genome and the *Arabidopsis *genome


**Linkage group**	**Markers**	**Position (cM)**	**BAC(s)**	**AGI range**	**No of gene Models predicted from *B. rapa *BAC**	**No. of Collinear gene models**

A1^a^	cnu_m618a cnu_m207a	54.1 54.1	KBrB080J22	At1g51410--At1g51420	27	9

A3^b^	cnu_m371a	79.0	KBrS012D09	At3g51400--At3g52980	27	20

A3^a, c^	nia_m057a cnu_m332a	63.9 67.2	KBrB058B22 KBrS008C11	At2g04540--At2g05755	43	23

A6^b^	cnu_m483a	69.2	KBrB044D19	At3g48950--At3g49210	31	17

A9^c^	cnu_m034a	7.7	KBrH097M21	At5g39760--At5g39880	27	15

A9^a^	cnu_m615a cnu_m157a	30.6 31.2	KBrH014M07	At2g20290--At2g20440	13	8

A9^b^	nia_m044a	36.4	KBrB072E02	At2g02170--At2g02950	20	10

^a^Confirmed by mapping a second marker.

^b^Identified by a single aligned BAC.

^c^Belong to our identified blocks.

Based on the 30 syntenic blocks, we detected 17 and 8 regions that were represented two and three times, respectively, within the *B. rapa *genome (Table 5). All redundantly represented regions were detected between different linkage groups, except one region represented three times, which was identified from two regions in A6 and one in A8 aligned to a 6.8-7.3 Mb region of *Arabidopsis *chromosome 1. The total sizes of the regions represented two and three times in the *B. rapa *genome were 18.9 Mb and 10.8 Mb, respectively, based on the sizes of the corresponding *Arabidopsis *regions. This result indicates that 29.7 Mb of the *Arabidopsis *genome corresponds to 70.2 Mb of the *B. rapa *genome. The detected redundant regions were smaller than 3.0 Mb (range: 0.1-3.0 Mb), indicating the scale of the maintained regions after hexapolyploidization. These genetically redundant regions were detected by the same cutoff value of sequence similarity, demonstrating that the regions had emerged simultaneously and undergone the same evolutionary events.

**Table 5:** Summary of the detected regions represented twice or three times in the *B. rapa *genome based on alignment to *Arabidopsis*


***Arabidopsis***			**Aligned***** B. rapa***	**Frequency in**	**Duplicated length in**
			**linkage group**	***B. rapa *genome**	***Arabidopsis *genome (Mb)**
**Chromosome**	**Aligned from (Mb)**	**Aligned to (Mb)**			

1	4.4	6.8	A6, A8	2	2.4
	6.8	7.3	A6, A6, A8	3	0.5
	7.3	7.4	A6, A8	2	0.1
	8.4	8.8	A8, A9	2	0.4
	8.8	10.6	A7, A8, A9	3	1.8
	10.6	10.8	A7, A9	2	0.2
	10.8	10.9	A7, A8, A9	3	0.1
	10.9	11.1	A8, A9	2	0.2

2	12.9	14.1	A3, A4	2	1.2
	14.1	16.3	A3, A4, A5	3	2.2
	16.3	18.6	A3, A5	2	2.3

3	2.5	4.5	A1, A5	2	2.0
	4.5	7.5	A1, A3, A5	3	3.0
	7.5	7.8	A3, A5	2	0.3
	8.1	8.9	A3, A5	2	0.8
	9.8	11.1	A5, A6	2	1.3
	19.5	20.1	A4, A9	2	0.6
	20.1	22.8	A4, A7, A9	3	2.7

4	8.7	8.8	A1, A3	2	0.1
	9.9	12.3	A3, A8	2	2.4
	12.3	17.8	A1, A3, A8	3	0.2
	17.8	18.4	A1, A8	2	0.6

5	1.6	4.2	A2, A10	2	2.6
	4.2	4.5	A2, A3, A10	3	0.3
	4.5	5.9	A2, A10	2	1.4

### Alignment of *B. rapa *linkage groups and karyotype

The *B*. *rapa *chromosomes are too small and compact to be distinguished by either morphological characteristics or the chromosome arm length ratio. In order to develop markers for complete karyotyping of these chromosomes, BAC clones genetically anchored on our reference genetic map were searched for repetitiveness by sequence similarities or FISH analysis. Consequently, 10 sets of nonrepetitive BAC clones (20 BACs) were selected to distinguish the 10 chromosomes and were fluorescence *in situ *hybridized on metaphase chromosomes (Table 6). The fluorescence signals of each set of BAC clones (red and green) were detected from each pair of 10 chromosomes (Additional file 3), confirming the utility of BACs in distinguishing the chromosomes. Six chromosomes hybridized by the selected BAC clones from A1, A3, A5, A6, A9, and A10 were recognized to be chromosomes 7, 2, 4, 5, 1, and 10, respectively, based on the following previously reported remarks: (1) the largest chromosome for chromosome 1, (2) the NOR-bearing chromosome for chromosome 2, (3) the 45S rDNA- and CentBr2-hybridized chromosome for chromosome 4, (4) the 45S rDNA-only-hybridized chromosome for chromosome 5, (5) the 45S rDNA- and 5S rDNA-hybridized chromosome for chromosome 7, and (6) the 5S rDNA-only-hybridized chromosome for chromosome 10 (Additional file 3). The four remaining chromosomes hybridized by BACs from A2, A4, A7, and A8 were assigned to chromosomes 3, 9, 6, and 8, respectively. This designation was based solely on the sizes of the four chromosomes, which were numbered from the largest to the smallest in accordance with chromosome morphology [24,31].

**Table 6:** Assignment of the linkage groups to chromosomes with karyotyping probes and remarks

**Karyotyping markers (BACs)**				**Results**	
	
**Linkage group**	**BAC ID**	**Location (cM)**	**FISH color**	**Remarks**^c^	**Anchored chromosome**
A1	KBrH003F06	43.5	red	45S rDNA, 5S rDNA	Chr 7
	KBrH003N18^b^	120.4	green		
A2	KBrB013N08^b^	37.9	red	None	Chr 3
	KBrB005J17	82.1	green		
A3	KBrB056I08	27.4	red	Nor-bearing Chromosome	Chr 2
	KBrS012D09	79.0	green		
A4	KBrH004D08^b^	81.6	red	None	Chr 9
	KBrB071M14	4.0	green		
A5	KBrH003E08^b^	95.0	red	45S rDNA, CentBr2	Chr 4
	KBrS004I08	21.0	green		
A6	KBrB076B03^b^	47.5	red	45S rDNA	Chr 5
	KBrH009H15	117.2	green		
A7	KBrB084H08	45.3	red	None	Chr 6
	KBrB021P15^b^	91.7	green		
A8	KBrB006A15^b^	4.9	red	None	Chr 8
	KBrH005C21	40.4	green		
A9	KBrH143F19^a^	93.9	red	The biggest Chromosome	Chr 1
	KBrH143K20^a^	22.4	green		
A10	KBrB012O13^b^	50.5	red	5S rDNA	Chr10
	KBrH053G06	11.7	green		

^a^[16]

^b^Selected for FISH as in Figure 3.

^c ^[22-24]

**Figure 3:**
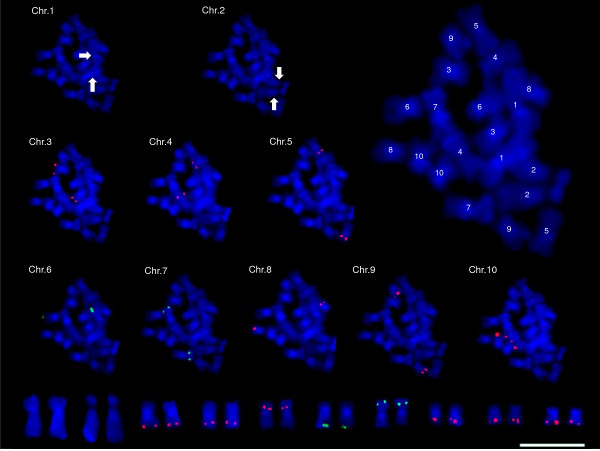
**FISH mapping of one karyotype marker from each of the eight sets on one set of *B. rapa *mitotic metaphase chromosomes**. The numbers on the magnified chromosomes represent chromosome numbers. The lowest row shows the karyotype of mitotic metaphase chromosomes with FISH patterns of the karyotype markers. White arrows indicate chromosomes 1 and 2, which were recognized by the following morphological remarks: the largest chromosome for chromosome 1 and the NOR-bearing chromosome for chromosome 2. Scale bar = 5 μm.

The 10 sets of BAC clones distinguishing each chromosome were defined as the definite karyotyping markers for the 10 chromosomes of *B. rapa*. In order to ensure the reliability of the markers, one BAC marker from each of the eight chromosome sets (chr 3-10; chromosomes 1 and 2 are obvious from their morphological remarks), was rehybridized onto one set of *B. rapa *chromosomes (Fig. 3). Consequently, all eight BACs were hybridized onto individual chromosome pairs, and chromosomes 1 and 2 were recognized by the morphological remarks (the largest chromosome for chromosome 1 and the NOR-bearing chromosome for chromosome 2), thus demonstrating the utility of the developed markers. The current orientations of four linkage groups (A1, A2, A3, A8) [16,27] are incorrect based on the orientation of chromosomes deduced from FISH signals (Fig. 3), and should be reversed.

## Discussion

### The second generation reference genetic map of *B. rapa*

A high density linkage map of *B. rapa *was constructed, resulting in an average marker density of one marker per 1.6 cM and the anchoring of 188 sequenced BAC clones. The SSR markers developed from these BAC sequences were validated by matching the sizes of the Chiifu alleles of the polymorphic band with the PCR product from the corresponding BAC clone. Approximately 57% of the markers on the map were of the SSR type, which are likely to be transferable to studies involving other *Brassica *species and populations. Moreover, the 188 sequenced BAC clones anchored on the reference genetic map will serve as a basis for integrating the physical map of *B. rapa *[32] with the reference genetic map.

Anchoring of the sequenced BACs onto the genetic map provided opportunities for aligning the reference map onto the *Arabidopsis *genome sequence on the basis of sequence similarity. Although *Arabidopsis *and *B. rapa *diverged approximately 20 million years ago [33], and underwent reshuffling during their respective evolutions, extensive collinearity was maintained between their genomes. On the basis of the 30 blocks of conserved synteny detected in this study, we illustrated possible contractions in the *B. rapa *genome. For example, in blocks 7 and 25, we estimated that 1 cM of the *B. rapa *map aligns with 1.2 Mb and 2.8 Mb of the *Arabidopsis *genome, respectively. In addition to the identified regions represented twice within the *B. rapa *genome (Fig. 1, Fig. 2, Table 3), the detected regions represented three times in the genome (Table 5) were similarly divergent in each case in that they were distinguished by the same level of sequence similarity. This supports the hypothesis that proposes the existence of a hexaploid ancestor in the evolution of *Brassica *species [1-6].

Most of the alignments between the linkage map and the *Arabidopsis *genome sequence are consistent with previously identified collinearity blocks [27,28]. These were initially inferred for *B. napus *using sequenced markers with homology to single *Arabidopsis *genes [27], and subsequently extended across a number of Brassicaceae [28]. However, our alignments are considerably more robust, as they exploit sequences of whole BAC clones containing homologies to multiple collinear *Arabidopsis *gene models. Further, the alleles present in the BAC clones can be matched to the mapped bands in the marker assays. This advantage overcomes the "noise" encountered in comparative genomics as a consequence of homologies to transduplicated gene fragments [6] and enabled us to identify seven previously unreported collinearity blocks. Two of the newly discovered collinearity blocks, represented by BAC clones KBrS008C11/KBrB058B22 and KBrH014M07, correspond to parts of blocks G and H, respectively, of the proposed Brassicaceae ancestral karyotype linkage group AK3 [28]. To date, these have been identified in only a single copy, and these newly discovered copies appear to represent one more of the three proposed paralogs of each that are expected to have arisen as a result of the proposed hexaploidy event in the ancestry of the *Brassica *species [1-6]. Block K, represented by KBrB072E02, also represents a third copy of the paralogs. Block M, not recognized in the A genome of *B. napus *[28], was identified. A newly discovered collinearity block represented by BAC clones KBrB080J22, KBrS012D09, and KBrH097M21 correspond to parts of blocks C, N, and S, respectively. These represent the fourth copies of the blocks to be identified, only three copies having being described by Schranz et al. [28]. We interpret the discovery of these blocks as an indication that supernumerary segmental genome duplications, as described for the genomic regions containing the *B. rapa *orthologs of the *Arabidopsis *gene *FLC *[5], could be common. Five of these blocks (D, G, H, H, and N) were recognized in the A genome of *B. juncea *[29], supporting our new findings.

### Integration of the reference genetic map with the 10 chromosomes of *B. rapa*

The twenty BAC clones used in this study were nonrepetitive and genetically anchored, thus allowing integration of the physical and genetic maps with each chromosome. As reported previously, comparative genetic mapping between *Arabidopsis *and *B. rapa *has revealed that collinear regions of the genomes are represented two or three times in *B. rapa *[5,34-36]. Jackson et al. [35] reported multiple duplications of the unique locus on *Arabidopsis *chromosome 2 (79 cM region) in the *B. rapa *genome by showing BAC FISH signals from four to six *B. rapa *chromosomes. In our study, some BACs were located at the loci represented twice (KBrB006A15, KBrB012O13, KBrB013N08, KBrB056I08, KBrB076B03, KBrH003E08, KBrH003N18, KBrH004D08) or three times (KBrH003F06, KBrH005C21, KBrB084H08) based on the *Arabidopsis *alignment data (Fig. 1, Fig. 2, Table 5). However, they were sufficiently divergent, showing unique hybridization and a single BAC FISH signal.

When the BrGSP was launched in 2003, the genome was assigned to participating countries by linkage groups, because at that time the chromosomes were not reconciled with the linkage groups. The previously reported karyotyping of *B. rapa *was based on chromosome length, and/or FISH patterns of repetitive DNAs [14,22-25]. From these studies, six chromosomes were distinguished unambiguously based on the chromosomal position of 45S rDNA, 25S rDNA, 5S rDNA, and centromeric repeats, whereas the remaining four chromosomes were ambiguous. Fukui et al. [22], Snowdon et al. [23], and Koo et al. [24] designated the recognized chromosomes to chromosomes 1, 2, 4, 5, 7, and 10 in association with chromosomes of the *Brassica s*pecies, corresponding to chromosomes 1, 2, 5, 4, 3, and 10 of Lim et al. [14,25], respectively. These discrepancies in assigning chromosome numbers are due to the specific characteristics of *Brassica *chromosomes, which are small and compact, causing different decisions on chromosome length order in the different studies, particularly with metaphase chromosomes. As described earlier, matching chromosomes in different studies in terms of chromosome length is sometimes difficult, and thus emphasizes the importance of the karyotype markers used in this study, which standardize the karyotype. Our chromosome nomenclature is consistent with earlier reports by Fukui et al. [22], and these have been reinforced by Snowdon et al. [23] and Koo et al. [24].

FISH has been used not only for chromosome identification [37] but also for merging genetic, physical, and chromosomal maps [38-42], thereby providing information about genome organization. The first integration of the cytogenetic and genetic linkage maps in *Brassica *species was achieved in CC genome species by FISH using 22 probes representing 19 loci of nine chromosomes [37]. In the present study, we assigned all 10 linkage groups of the *B. rapa *reference genetic map to each of the 10 chromosomes and corrected the orientation of the linkage groups by FISH. The 20 cytologically mapped loci will serve as the basis for integration of the genetic, physical, and chromosomal maps of *B. rapa *in the multinational BrGSP.

## Conclusion

We constructed a second generation reference linkage map of *B. rapa*, which has an average marker density of 1 marker per 1.6 cM and to which 188 sequenced BAC clones are anchored. Anchoring of the sequenced BACs onto the reference genetic map provided opportunities for aligning the map onto the *Arabidopsis *genome sequence on the basis of sequence similarity. We detected 30 blocks of conserved synteny between the *B. rapa *and *Arabidopsis *genomes, illustrating rearrangement events with a trace of hexapolyploidy differentiating these genomes. Most of these were consistent with previously reported collinear blocks; however, we were able to identify seven regions as individual BAC clones or a pair of overlapping BAC clones, representing previously unreported collinearity blocks. One of these represents a previously "missing" block under the hypothesis of whole genome triplication, and three of the remaining blocks represent a supernumerary segment under the same hypothesis.

Nonrepetitive and genetically anchored BAC clones allowed integration of the genetic map with the cytogenetic map by developing definite karyotype markers. This is the first unambiguous alignment of the linkage map with the 10 *B. rapa *chromosomes. We envision that the genetic map and analysis presented here will serve as a basis for integrating the genetic, physical, and chromosomal maps of *B. rapa *in the multinational BrGSP as well as for studies on polyploidization and speciation in the genus *Brassica*.

## Methods

### SSR marker development

Five hundred and twenty one sequenced BACs of *B. rapa *ssp. *pekinensis *generated by the KBGP [12] were collected from GenBank [43]. SSRs were identified from the annotated BACs with SPUTNIK [44] using the parameters described previously by Hong et al. [15]: (i) SSRs were determined to be positive if the repeats were ≥ 12 bp for mononucleotide, dinucleotide, and trinucleotide repeats, ≥ 16 bp for tetranucleotide repeats, and ≥ 20 bp for pentanucleotide repeats; and (ii) no variations (mutations) in repeat motifs were permitted

SSR loci of ≥ 18 bp, as described in the preceding, were chosen for marker development. One to three SSR primer sets per BAC were designed using Primer3 software [45] from the flanking sequences of the targeted SSR loci. The primer design criteria were as follows: 100-400 bp of amplicon size, 55-63°C of Tm, >35% of GC contents, and >18 bp of primer length. Totally of 749 primer sets were designed from 367 BAC sequences (Additional file 1). To develop SSR markers, polymorphisms between the parental lines (Chiifu-401-42 and Kenshin-402-43) [16] were evaluated by PCR amplification of the parental genomic DNA using the designed primer sets. PCR products were separated on 6% polyacrylamide gels and visualized by using a silver staining kit (Bioneer, Daejeon, Korea). Allele matching was performed by analysis of PCR products from genomic DNA of Chiifu-401-42 and the BAC clone from which primers were designed. Mapping was conducted only for markers where the size of the polymorphic band corresponding to the Chiifu allele matched the size of the PCR product from the BAC.

PCR amplification was carried out in 10 μl volumes, containing 0.5 units of *Taq *polymerase (Bioneer, Daejeon, Korea), 5 pmol of each primer, 250 μM dNTPs, 2.0 mM MgCl_2_, 1× *Taq *buffer, and 10 ng of genomic template DNA. The PCR profile was as follows: 5 min at 95°C, followed by 30-35 cycles with 30 s of DNA denaturation at 94°C, 30 s of annealing at the appropriate temperature, and 60 s of extension at 72°C, and final extension at 72°C for 7 min. PCR was carried out in a Bioneer thermal-cycler (Bioneer, Daejeon, Korea). We followed the locus nomenclature form used in previous reports by Choi et al. [16] which is based on the De Vicente et al. [46] format. The institute codes refer to the laboratory where each primer was screened; those used in this study were 'cnu' for Chungnam National University and 'nia' for the National Institute of Agricultural Biotechnology.

### Genetic mapping

Previously reported plant materials, including two parental inbred lines and a 78-line DH population (CKDH) [16], were used for genetic mapping. Plant genomic DNA was isolated from fresh leaf material according to the procedure used by Guillemaut and Maréchal-Drouard [47].

Markers that were reproducibly polymorphic between parent lines were genotyped in the DH population. Linkage analysis and map construction were performed using JoinMap version 4.0 [48,49]. Linked loci were grouped in the LOD grouping threshold range of 3.8-5.0, and linkage groups were assigned as A1 to A10, corresponding to the previously reported initial version map [16] of this species. Locus order within the LOD grouping was generated for each linkage group using a recombination frequency below 0.45 and an LOD score above 0.5 for all marker pairs within each linkage group. A "ripple" procedure was performed after the addition of each marker and the "jump" thresholds were set to 5. Recombination frequencies were converted to centiMorgans (cM) with Kosambi's method for map-distance calculation [50].

### Aligning the linkage map with the Arabidopsis genome sequence

All 188 sequenced seed BACs [12] anchored to the genetic linkage groups were used for the genome alignment. Repeat sequences in both the *B. rapa *BACs and the *A. thaliana *genomic sequences [51] were masked by RepeatMasker [52] and cross-matched [53] with our repeat database collected from the TIGR plant repeat database [54] and Repbase [55]. Each of the *B. rapa *BACs were aligned to the *A. thaliana *genome sequence using BLASTZ [56] with parameters of [B = 0, C = 2, K = 2200]. The most highly conserved regions (HCRs) for the alignments between the species were identified by the following criteria: (i) total length of the aligned regions in HCRs between the species should be ≥ 5 kb and (ii) the number of aligned regions in HCRs should be more than three. To reinforce the method for confirming HCRs between the two species, *B. rapa *genes in the BAC sequences predicted by GenScan [57] and were compared with *A. thaliana *genes by BLAST X [58] with a cutoff value of 1e-10.

### Chromosome preparation

Seeds from *B. rapa *ssp. *pekinensis *cv. Chiifu-401-42 were germinated on moist filter paper in petridishes at 25°C for 48 hr. Root tips were sampled and prepared for FISH as previously described by Koo et al. [24] with the following modifications: root tips were excised and incubated in 2% cellulase (Sigma, St. Louis, USA), 1.5% macerozyme (Sigma, St. Louis, USA), 0.3% pectolyase (Sigma, St. Louis, USA), and 1 mM EDTA (pH 4.2).

### Fluorescence *in situ *hybridization probe preparation

Two BACs from each of the 10 linkage groups (Table 6) were selected as FISH probes to anchor the linkage groups to the chromosomes as well as to distinguish individual chromosomes of *B. rapa *ssp. *pekinensis *cv. Chiifu-401-42. The BAC clone, KBrH077I01, containing 176 bp of centromeric tandem repeats, was used as the CentBr2 probe (unpublished data). BAC DNA was extracted by a modified alkaline lysis method [59] with the following changes: BAC clones were cultured for 18 hr in 3 ml of LB media containing 12.5 μg/ml of chloramphenicol and harvested by centrifugation followed by decanting of the media. Cell pellets were resuspended in 200 μl of solution I (50 mM Tris·HCl, 10 mM EDTA [pH 8.0], 100 μg/ml RNase A) followed by shaking using a vortex. Lysis and neutralization were achieved by adding 200 μl of solution II (0.2 M NaOH, 1% SDS) and 200 μl of solution III (3.0 M potassium acetate [pH 5.5]) into the sample plates without a lysis incubation time. The lysates were then cleared and precipitated, followed by 70% ethanol washing. Each precipitated DNA pellet was re-suspended in 25 μl of 1 mM Tris (pH 8.0). BAC DNAs were labeled with biotin-16-dUTP using nick translation kits (Roche, Basel, Switzerland). The reaction was carried out at 15°C for 90 min followed by reaction blocking by the addition of 2 μl of 0.5 M EDTA. The reaction products were purified by ethanol precipitation.

The 45S and 5S rDNAs were amplified from the total genomic DNA of *B. rapa *by PCR using the following primer sets: 5'-TACCTGGTTGATCCTGCCAG-3' (forward) and 5'-TTGTCACTACCTCCCCGTGT-3' (reverse) for 45S rDNA [60]; 5'-GATCCCATCAGAACTTC-3' (forward) and 5'-GGTGCTTTAGTGCTGGTAT-3' (reverse) for 5S rDNA [61]. The PCR cycling reaction was carried out in 100 μl volumes containing 2.5 units of *Taq *polymerase (Takara, Kyoto, Japan), 5 pmol of each primer, 250 μM dNTPs, 1× PCR buffer, and 10 ng of genomic template DNA. The amplifying PCR cycle was as follows: 35 cycles with 1 min of DNA denaturation at 94°C, 1 min of annealing at 55°C, and 1 min of extension at 72°C, followed by a 10 min final extension at 72°C. The amplified products were purified using a QIAquick gel extraction kit (Qiagen, Hilden, Germany). Labeling of the 45S and 5S rDNA was performed by a PCR cycling reaction in a 100 μl volume containing 2.5 units of *Taq *polymerase (Takara, Kyoto, Japan), 5 pmol of primer, 200 μM dATP, dCTP, dGTP, 140 μM dTTP, 60 μM digoxigenin-dUTP (Roche, Basel, Switzerland), 1× PCR buffer, and 10 ng of template rDNA. The labeling PCR cycle was identical to the amplifying cycle described previously.

### Fluorescence *in situ *hybridization

FISH was carried out as described by Koo et al. [24] using 50 ng of labeled probes (BAC DNA, 45S rDNA, 5S rDNA, and CentBr) per slide. Briefly, chromosomal DNA on the slides was denatured with 70% formamide at 70°C for 2 min, followed by dehydration in 70, 85, 95, and 100% ethanol at -20°C for 3 min each. The probe mixture, containing 50% formamide (v/v), 10% dextran sulfate (w/v), 5 ng/μl salmon sperm DNA, and 500 ng/μl of labeled probe DNA, was denatured at 90°C for 10 min and kept on ice for 5 min. A 20 μl volume of the probe mixture was applied to the denatured chromosomal DNA and covered with a glass coverslip. Slides were then hybridized in a humid chamber at 37°C for 18 hr followed by standard washing (50% formamide for 10 min at 42°C, 2× SSC for 5 min at 42°C, and 4 × SSC/0.2% Tween-20 for 5 min at 42°C) and blocking (5% BSA/4 × SSC/0.2% Tween-20 for 10 min at RT). Probes were detected with avidin-FITC and anti-digoxigenin Cy3 (Roche, Basel, Switzerland). Chromosomes were counterstained using 1 μg/ml of DAPI (Vector Lab, Burlingame, USA). The signals were detected with a Cooled CCD Camera, CoolSNAP (Photometrics, Tucson, USA), and images were processed with software (Meta Imaging Series, version 4.6; Molecular Devices, Downingtown, USA) using a Leica epi-fluorescence microscope equipped with FITC-DAPI two-way or FITC-Rhodamine-DAPI three-way filter sets (Leica, Wetzlar, Gemany). The final printed images were prepared with Adobe Photoshop, version 8.0.

Reprobing wash was performed with 4 × SSC/0.2% Tween-20 for 30 min at 37°C, followed by dehydration in 70, 85, 95, and 100% ethanol at -20°C for 2 min each.

## Authors' contributions

HRK designed the study, carried out the marker development, participated in the FISH analysis, analyzed and interpreted all data, and drafted the manuscript. SRC carried out genetic mapping, participated in the comparative anlaysis, and drafted the manuscript. JB performed the FISH experiment and participated in drafting the manuscript. CPH carried out the SSR analysis, participated in the comparative analysis, and drafted the manuscript. SYL, MJH, DVN, and MJ participated in the marker survey and genotyping. BSP and JWB participated in the design of the study and helped to draft the manuscript. IB participated in the synteny analysis and interpretation of alignment data, and drafted the manuscript. YPL conceived the study, participated in its coordination, and helped to draft the manuscript. All authors read and approved the final manuscript.

## Supplementary Material

Additional file 1 Details of the 367 BAC sequence derived SSR primers including 191 newly mapped SSR markers. Click here for file

Additional file 2 Details of the alignment between the *Brassica rapa *reference genetic map and the *Arabidopsis *genome sequence. Click here for file

Additional file 3 **Fluorescence *in situ *hybridization (FISH) mapping of 45S rDNA, 5S rDNA, CentBr2, and karyotyping markers (BACs) on the mitotic metaphase chromosomes of *Brassica rapa***. White arrows indicate the pair of chromosomes hybridized by each set of karyotyping markers (BACs). (O) and (X) indicate 'hybridized' and 'not hybridized' respectively, on the chromosome pair recognized by each set of karyotyping markers. Scale bar = 5 μm. Click here for file
