# Supplementary material for: Sequenced BAC anchored reference genetic map that reconciles the ten individual chromosomes of Brassica rapa
Source: BMC Genomics. 2009 Sep 15;10:432. doi: 10.1186/1471-2164-10-432 (PMC2761421; doi:10.1186/1471-2164-10-432)
Supplement: Additional file 3 — Fluorescence in situ hybridization (FISH) mapping of 45S rDNA, 5S rDNA, CentBr2, and karyotyping markers (BACs) on the mitotic metaphase chromosomes of Brassica rapa. White arrows indicate the pair of chromosomes hybridized by each set of karyotyping markers (BACs). (O) and (X) indicate 'hybridized' and 'not hybridized' respectively, on the chromosome pair recognized by each set of karyotyping markers. Scale bar = 5 μm. [file 1471-2164-10-432-S3.pdf]

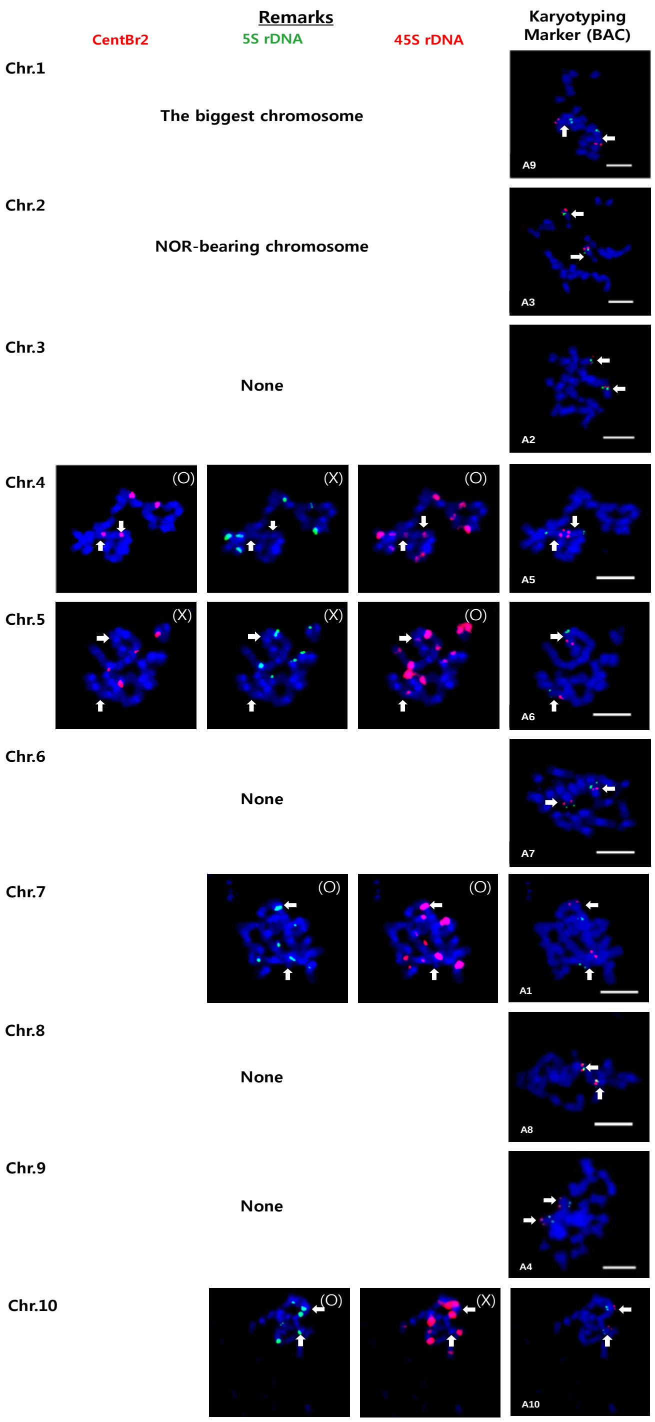

Additional file 3 - Fluorescence *in situ* hybridization (FISH) mapping of 45S rDNA, 5S rDNA, CentBr2, and karyotyping markers (BACs) on the mitotic metaphase chromosomes of *Brassica rapa*. White arrows indicate the pair of chromosomes hybridized by each set of karyotyping markers (BACs). (O) and (X) indicate 'hybridized' and 'not hybridized' respectively, on the chromosome pair recognized by each set of karyotyping markers. Scale bar = 5 μm.
